# Enhancement of the Inner Foveal Response of Young Adults with Extended-Depth-of-Focus Contact Lens for Myopia Management

**DOI:** 10.3390/vision8020019

**Published:** 2024-04-14

**Authors:** Ana Amorim-de-Sousa, Rute J. Macedo-de-Araújo, Paulo Fernandes, José M. González-Méijome, António Queirós

**Affiliations:** 1 Clinical and Experimental Optometry Research Lab (CEORLab), School of Science, University of Minho, 4710-057 Braga, Portugal; 2 Physics Center of Minho and Porto Universities (CF-UM-UP), 4710-057 Braga, Portugal

**Keywords:** myopia control, contact lens, peripheral refraction, gf-mfERG, electrophysiology

## Abstract

Background: Myopia management contact lenses have been shown to successfully decrease the rate of eye elongation in children by changing the peripheral refractive profile of the retina. Despite the efforts of the scientific community, the retinal response mechanism to defocus is still unknown. The purpose of this study was to evaluate the local electrophysiological response of the retina with a myopia control contact lens (CL) compared to a single-vision CL of the same material. Methods: The retinal electrical activity and peripheral refraction of 16 eyes (16 subjects, 27.5 ± 5.7 years, 13 females and 3 males) with myopia between −0.75 D and −6.00 D (astigmatism < 1.00 D) were assessed with two CLs (Filcon 5B): a single-vision (SV) CL and an extended-depth-of-focus (EDOF) CL used for myopia management. The peripheral refraction was assessed with an open-field WAM-5500 auto-refractometer/keratometer in four meridians separated by 45° at 2.50 m distance. The global-flash multifocal electroretinogram (gf-mfERG) was recorded with the Reti-port/scan21 (Roland Consult) using a stimulus of 61 hexagons. The implicit time (in milliseconds) and response density (RD, in nV/deg^2^) of the direct (DC) and induced (IC) components were used for comparison between lenses in physiological pupil conditions. Results: Although the EDOF decreased both the HCVA and the LCVA (one and two lines, respectively; *p* < 0.003), it still allowed a good VA. The EDOF lens induced a myopic shift in most retinal areas, with a higher and statistically significant effect on the nasal retina. No differences in the implicit times of the DC and IC components were observed between SV and EDOF. Compared with the SV, the EDOF lens showed a higher RD in the IC component in the foveal region (*p* = 0.032). In the remaining retinal areas, the EDOF evoked lower, non-statistically significant RD in both the DC and IC components. Conclusions: The EDOF myopia control CL enhanced the response of the inner layers of the fovea. This might suggest that, besides other mechanisms potentially involved, the central foveal retinal activity might be involved in the mechanism of myopia control with these lenses.

## 1. Introduction

Currently, there are several commercially available options for myopia progression management, including ophthalmic lenses such as spectacles and contact lenses (CLs) [1]. The latter were among the first corrective tools developed and applied to myopia management, with several different optical designs already available. Contact lenses for myopia management have proven their safety [2] and effectiveness in decreasing the rate of eye elongation in children [3,4]. With a single-vision optical element, the peripheral refractive profile of the myopic eye tends to be hypermetropic. Myopia control optical strategies intend to produce a myopic shift in peripheral refraction [4].

Orthokeratology is one of the most common methods to induce myopic changes in peripheral refraction. This treatment is a safe and effective method to control myopia progression with a retention rate between 37 and 56% in axial length growth over two years [5]. Within the same period, soft CLs with multifocal designs have also shown satisfactory results, with retention between 0.29 D and 0.54 D in myopic refraction and up to 0.39 mm for axial length [6,7,8,9,10].

Soft CLs with extended-depth-of-focus (EDOF)—Mylo, Mark’ennovy ©, Spain—and dual-focus (DF)—MiSight, Coopervision, The Cooper Companies Inc., UK, and Defocusing Incorporated Soft Contact (DISC), St. Shine Optical Co. Ltd., Taiwan, China—profiles are currently used for myopia management [7,10,11]. While DF lenses consist of alternating distance and +2.00 to +2.50 D near-vision rings surrounding the center-distance zone [6,7,11,12], the EDOF design allows a through-focus up to +1.75 D from the distance refraction due to a non-monotonic and aperiodic power profile incorporating high-order aberrations, mainly spherical aberration [9,13]. These CL designs showed comparable results in visual performance, optical quality changes and accommodative changes [9,11,12,14].

Although these multifocal contact lenses are commercially available as a myopia management treatment, the physiological mechanisms that enable slowing down the eye growth rate in children are not yet well understood. Some electrophysiological studies revealed a decrease in the retinal electrical activity of myopic eyes [15,16,17,18,19,20], possibly related to the impaired function of the ON pathway [15,21]. Luu et al. [22] reported a uniform reduction in the adults retinal activity of myopes compared to emmetropes, significantly correlated with the magnitude of myopia. In children, a study by Li et al. [23] observed subclinical decreased central inner retinal activity before the development of myopia, which was significantly related to later refractive errors. 

The retinal electrical activity of different areas changes differently with myopic defocus, with a possible higher influence in the peripheral retina [24,25,26]. While the response of the central retinal area decreases with myopic defocus [25], some studies showed an increase in the parafoveal retinal response while hyperopic defocus decreased the response amplitude of the outer retinal layers of the parafovea [26,27]. Chin et al. [27] reported similar results for hyperopic (−2 D) and myopic (+2 D) defocus. Myopic defocus increased the DC amplitude at more peripheral rings, and hyperopic defocus decreased it, while in central retinal areas, the amplitude increased with both types of defocus. For the IC response, there were no clear trends for either positive or negative defocus. These findings are reflected in the results of a study by Fung et al. [28] using dual-focus contact lenses with concentric in-focus and positive defocus rings. Fung et al. observed an enhancement in the retinal response in the central and para-macular regions. Although this enhancement (increase in amplitude) was observed in both the DC and IC components, only that originating from the inner layers (the IC component) was significant with a dual-focus contact lens with +7.50 D addition [28]. The results from these works suggest the existence of a decoding system for optical defocus located in peripheral areas beyond the fovea. 

The present study aimed to investigate possible global and local short-term changes in the retinal response driven by the optical changes induced by extended-depth-of-focus CLs used for myopia management, compared to material-matched single-vision contact lenses. Regarding the effectiveness of myopia control strategies on the rate of eye growth and assuming that there is a local retinal mechanism of defocus detection, it was hypothesized that it is likely to detect changes in the electrophysiological response of the retina with both myopia management contact lenses.

## 2. Materials and Methods

### 2.1. Study Design

This masked cross-sectional study analyzed possible differences in the retinal response at different locations and cellular layers between a single-vision (SV) CL and one CL for myopia management. This study was conducted at CEORLab (Clinical and Experimental Optometry Research Laboratory, University of Minho, Braga, Portugal) with the approval of the Ethics Committee for Research in Life and Health Sciences (CEICVS) of the Ethics Council of the University of Minho (CEUMinho). The protocol conception followed the guidelines of the Declaration of Helsinki, and the participants were duly informed about the study procedures and signed an informed consent form.

### 2.2. Recruitment

For the recruitment, this study was disseminated among the academic population of the University of Minho. To be part of our study, the volunteers had to meet the following inclusion criteria: age between 18 and 35 years, myopic spherical equivalent between −0.75 D and −6.00 D, astigmatism not more than 1.00 D (included), absence of loss of ocular media transparency or other ocular pathologies, as well as no systemic health problems or medication potentially causing changes in the visual system. Exclusion criteria included individuals with the best corrected visual acuity worse than 0.2 logMAR, anisometropia, previous surgical eye intervention or any past myopia management strategy. Before enrollment, volunteers who met the requirements had to sign an informed consent form.

### 2.3. Protocol

After an enrollment visit (1st visit), the participants were required to undergo a 2nd visit to the laboratory (2nd visit) to minimize the impact of tiredness on the measurements, as follows: 

1st visit: To ensure compliance with all inclusion criteria, participants underwent an ophthalmic examination of both eyes (refraction, visual acuity, slit-lamp and fundus observation). The spectacle’s refraction that provided the best visual acuity was registered. The CLs were from a custom-made manufacturer, so the topographic data (flat and steep curvature radius—Kflat and Ksteep) and the horizontal visible iris diameter (HVID) were acquired. 

2nd visit: High- and low-contrast visual acuity, aberrometry, peripheral refraction profile and an electroretinography test were acquired, in this order, with the single-vision CL (SV, control) and the myopia management (test) CL on the same visit. When the inclusion requirements were met, all measurements were taken in both eyes. The measurement order between CLs was randomly selected through a Microsoft Excel^®^ function. The measurements were performed 30 min after CL insertion in both control and test conditions, which exceeds the time of defocus detection in the human eye [29]. The second CLs were inserted 5 min after the removal of the previous ones. The participants were not aware if they were using the control or test CLs.

### 2.4. Contact Lenses

Two commercially available soft CLs of silicone hydrogel (Filcon 5B) with distinct designs were used: a single-vision (SV) CL and an extended-depth-of-focus CL design used in children for myopia control [9,10]. The SV CL was used as the control for its myopia management counterpart to account for the potential influence of the material in electrophysiological recordings [30]. The distance correction power of the two CLs was the same, considering the spherical equivalent refraction of each participant. The centration of the contact lenses was checked using corneal topography, but the impact of those data is not included in the present study to avoid overloading it with information.

The two CLs were monthly disposables with customized parameters (diameter, curvature radius and eccentricity). The manufacturer conducted the calculus for manufacturing the CLs with the appropriate data (K_flat_, K_steep_, HVID and eccentricity values) assessed in the 1st visit with the corneal topographer Medmont E300 (Medmont Pty. Ltd., Melbourne, Australia). The base curvature and diameter of the two contact lenses (SV and EDOF) for the same subject were the same. Additional technical specifications for the CLs are shown in Table 1.

**Figure 1:**
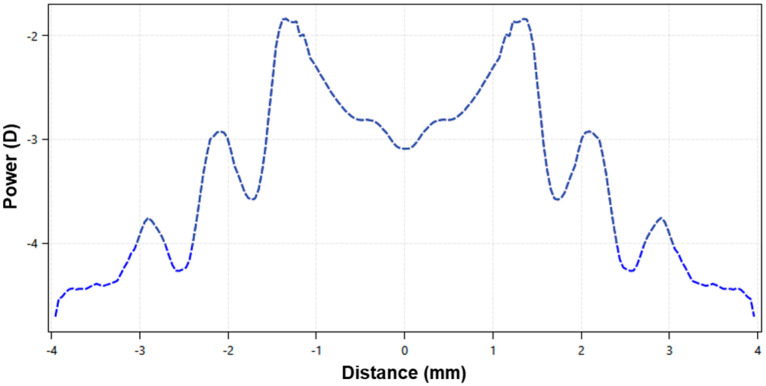
Power profile distribution, in diopters, of a −3.00 D extended-depth-of-focus contact lens for myopia management, with up to +1.75 D of add power.

The EDOF lens has a non-monotonic and aperiodic power profile across the optic zone, with smooth transitions between the multiple zones (Figure 1). The lens design incorporates high-order aberration, especially spherical aberration, to achieve a through-focus up to +1.75 D from the distance refraction (Figure 1). This provides good optical quality within a single-elongated focal point anterior to the retina while rendering the optical quality of the image points posterior to the retina poorer [9,13].

### 2.5. Visual Acuity

The monocular and binocular visual acuity was measured in logMAR units using the Early Treatment Diabetic Retinopathy Study (ETDRS) chart at 4 m distance from the observer. The high (100%)- and low (10%)-contrast visual acuities (HCVA and LCVA, respectively) were assessed with a room luminance at photopic levels (85 cd/m^2^, Luminance meter LS-150, Konica Minolta, Osaka, Japan).

### 2.6. Aberrometry

The higher-order aberrations (HOAs) were acquired with the IRx3 Hartmann–Shack aberrometer (ImaginEyes, Orsay, France) under mesopic and non-dilated pupil conditions, prior to peripheral refraction. For the acquisition, the participants were asked to blink twice, keeping their eyes open during the measurement while fixating on the letter “E” inside the aberrometer. Three measurements were recorded with each lens and averaged for analysis. In each measurement, it was ensured that the natural pupil diameter was superior to 5 mm. The coefficients of the Zernike polynomial (in microns, µm) of the 4th ($C_{4}^{0}$) and 6th ($C_{6}^{0}$) order spherical-like HOAs (SA) and the 3rd and 5th order coma-like HOAs for vertical ($C_{3}^{-1}$ and $C_{5}^{-1}$, respectively) and horizontal ($C_{3}^{1}$ and $C_{5}^{1}$, respectively) coma, as well as the root mean square of the total HOAs (total RMS), were considered for analysis and extracted for a 5 mm pupil diameter. 

### 2.7. Peripheral Refraction

**Figure 2:**
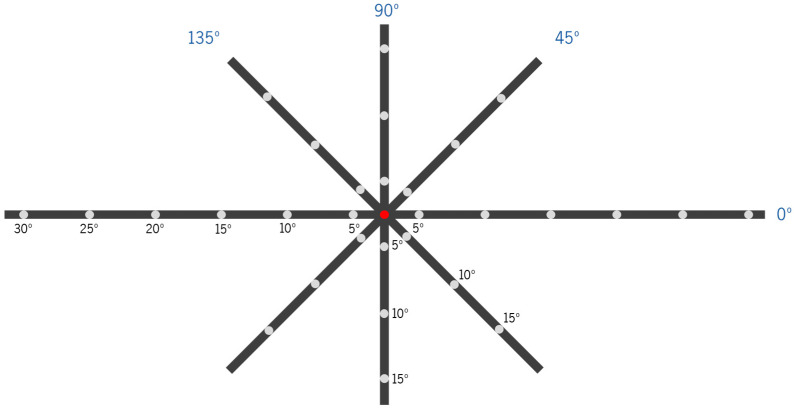
Scheme of the fixation device for different retinal eccentricity projections for the measurement of the peripheral refraction at 2.50 m with eye rotation (head fixed in the auto-refractometer’s chin cup) in four meridians (horizontal (0°), 45°, vertical (90°) and 135°, all in dark blue). Each fixation point is separated by 5° of retinal eccentricity, considering the viewing distance. Subjects were asked to look at a red light lit at each point for the refraction measurement at the respective position. In the scheme, the condition of on-axis refraction measurement (central-point fixation (0°) of eccentricity) is illustrated.

On- and off-axis objective refraction were measured with the two CLs, using a WAM-5500 auto-refractometer/keratometer (GrandSeiko Co., Ltd., Hiroshima, Japan). Measurements were performed using a fixation cross with LED lights at 2.5 m from the observers, whose location corresponded to retinal eccentricities in steps of 5°. Measurements were obtained for the horizontal (from 5 to 30° nasal, center, and from 5 to 30° temporal), vertical (from 5 to 15° superior and from 5 to 15° inferior) and oblique meridians at 45°, 135°, 225° and 315° (from 5 to 15°), as represented in Figure 2. These points were converted and analyzed according to the nasal (N), temporal (T), superior (S), inferior (I), superior–nasal (SN), superior–temporal (ST), inferior–nasal (IN) and inferior–temporal (IT) projections into retinal eccentricities. The subjects would initially place their forehead and chin in the corresponding rest places, aligned with the central point (center, 0° eccentricity, red spot in Figure 2), and look to each point according to the researcher’s needs without moving their heads. The auto-refractometer took five quick automatic measures of the sphere, cylinder (in steps of 0.01 D) and axis (in steps of 1 degree). The average of the five values was automatically recorded in an Excel sheet (Microsoft^®^ Office Proofing Tools, 2018 Microsoft Corporation, Los Angeles, CA, USA) using software directly connected to the auto-refractometer. A pupil diameter >4 mm is required for off-axis measurements with the GrandSeiko auto-refractometer. The room illumination allowed us to achieve this requirement in all subjects.

The clinical refraction (expressed as a sphere, negative cylinder and axis) was converted into the vectorial components M, J0 and J45, calculated [31] as follows in Equations (1)–(3):

(1) $$M=Sphere+\frac{Cylinder}{2}$$

(2) $$J0=\frac{Cylinder}{2}\times \cos\left(2axis\right)$$

(3) $$J45=\frac{Cylinder}{2}\times \sin\left(2axis\right)$$

For data analysis, the spherical equivalent (M, in diopters) and the tangential (FT′) and the sagittal (FS′) foci (Equations (4) and (5), respectively) were used [32]. The relative peripheral refraction (RPR) of each component (M, FT′ and FS′) was calculated for each off-axis measurement by subtracting the on-axis value.

(4) $$FT^{\prime }=M-J0$$

(5) $$FS^{\prime }=M+J0$$

### 2.8. Electroretinography: Global-Flash Multifocal Electroretinogram

**Figure 3:**
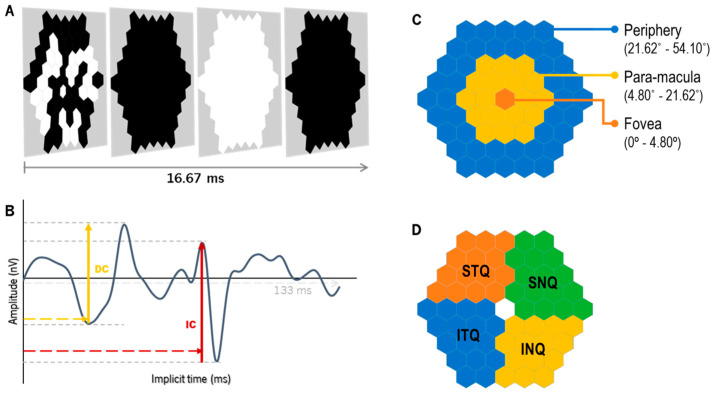
The global-flash multifocal ERG (**A**) stimulation is divided into four frames: the first frame presents the m-sequence pattern, followed by a dark frame, a global-flash frame and a second dark frame. After the recording, the wave response (**B**) with two defined components can be depicted: the direct component (DC), which mirrors the activity of the outer-to-middle retina, and the induced component (IC), with the main influence of the inner retinal layers. (**C**) Retinal eccentric areas with hexagon groups as fovea (from 0° to 4.80°, in orange), para-macula (from 4.80° to 21.62°, in yellow) and periphery (from 21.62° to 54.10°, in blue). (**D**) represents the grouped areas of analysis by quadrants, as follows: inferior–nasal (INQ, in yellow), superior–nasal (SNQ, in green), superior–temporal (STQ, in orange) and inferior–temporal (ITQ, in blue).

The response of the global-flash multifocal electroretinogram (gf-mfERG) [33] was recorded with the Retiport/scan 21 (Roland Consult, Germany). The stimulus was a sequence of a multifocal pattern flash frame composed of 61 black and white hexagons scaled with eccentricity (hexagon distortion factor of 1:4 from center to periphery), flickering according to a pseudorandom binary m-sequence, followed by a dark frame, a global-flash frame and a second dark frame (Figure 3A). The pattern stimulus was presented on a 19-inch LCD monitor (ProLite B1980SD, iiyama, Nagano, Japan) at a viewing distance of 33 cm (total field of view approximately 54.1°) with a frame rate of 60 Hz, resulting in a curve length similar to the one depicted in Figure 3B. The total recording time per lens was 3 min and 40 s. The mean luminance of the stimulus was 220.52 ± 1.54 cd/m^2^ for the white hexagons and 1.37 ± 0.08 cd/m^2^ for the black hexagons (Luminance meter LS-150, Konica Minolta, Osaka, Japan). The mean room illuminance at viewing distance was 154.83 ± 0.52 lux (Illuminance meter T-10A, Konica Minolta, Osaka, Japan).

The recording procedure followed the mfERG guidelines from the International Society for Clinical Electrophysiology of Vision (ISCEV) [34,35], except for pupil dilation. Before starting the acquisitions, the participants were prepared as in previous studies [16,30,36,37], using a Dawson–Trick–Litzkow (DTL-plus) electrode as the active electrode. The total preparation time ranged between 7 and 12 min. As participants were using CLs for myopia correction, additional rimless ophthalmic lenses of +3 D were placed at the anterior focal plane of each eye to avoid the accommodation effect at the viewing distance (33 centimeters). Safety and quality parameters were checked as in previous studies: impedance less than 10 kOhm, real-time response check, artifacts and behavior of the participants—blinking and loss of red cross fixation—through a built-in infrared camera of the software. This built-in camera also allows for monitoring pupil fluctuations during recordings. The gf-mfERG recordings were obtained monocularly in binocular viewing conditions. All measurements were performed with the pupil under physiological conditions (non-pharmacological dilation) to ensure that the experiments were made in conditions closer to daily conditions [14,38]. This also allows for the prevention of the possible influence of the mydriatic agent on the ERG response [39]. To ensure stable lighting conditions, pupil size was measured before and after each recording using an infrared pupilometer (VIP-200, NeurOptics, California, USA), in the same illuminance and luminance conditions as the recordings, while the subject was looking at the stimulation pattern on the screen.

The gf-mfERG response curves were analyzed for the total retinal response of the retina (SUM, from 0° to 54.10° eccentricity) and different retinal regions, according to eccentricity (fovea (from 0° to 4.80°), para-macula (from 4.80° to 21.62°, including parafovea and perifovea) and periphery (from 21.62° to 54.10°), Figure 3C) and quadrants (inferior–nasal (INQ), superior–nasal (SNQ), superior–temporal (STQ) and inferior–temporal (ITQ), Figure 3D). For each response curve, the implicit time of the peaks (in milliseconds, ms) and the response density (RD, in nano-Volts per square degree, nV/deg^2^) of the direct and induced components (DC and IC, respectively) were analyzed (Figure 3B). The parameters obtained with the SV and EDOF lenses were compared, as described below in the Statistics section.

### 2.9. Statistics

For the estimation of the sample size, the statistical significance (*p*-value) was set at 0.05 and a statistical power of at least 0.80, with a two-tails effect considering a coefficient variation of at least 20% in the mfERG response density between single-vision and myopia control CLs [30], superior to the repeatability of 15% of the RETIscan device found by Mazinani et al. [40]. GPower 3.1 software showed a minimum sample size of 4 eyes to fulfill those requirements.

The IBM SPSS Statistics v28.0 (IBM Inc., IL, USA) software was used for the statistical analysis. The normality of data distribution was first checked with the Shapiro–Wilk test (sample size < 30). The paired comparisons were conducted with a *t*-test or the Wilcoxon test, according to data distribution. The comparisons between SV and EDOF for gf-mfERG and peripheral refraction were analyzed using a two-way ANOVA (univariate analysis of variance) with post hoc Bonferroni corrections. The correlation between gf-mfERG and the relative peripheral refractive error was analyzed through Pearson’s or Spearman’s correlations, according to the nature of data distribution. Data are represented as the mean ± standard deviation (SD) and 95% confidence interval (CI), or as median [variance], considering the normality of the data. In some cases, the median values are followed by the interquartile range (IQR) for clarity as median [Q3-Q1], where Q3 corresponds to the 75th percentile (upper quartile) and Q1 corresponds to the 25th percentile (lower quartile).

## 3. Results

### 3.1. Sample Characteristics and Lens Centration

A total of 20 subjects were recruited for this study, but only 16 signed the consent form and attended the two visits. One participant was withdrawn due to inconsistencies in several variables. The right and left eyes did not reveal statistically significant differences. Consequently, one eye of each of the 16 remaining subjects was randomly chosen for data analysis, except for two subjects who only met the astigmatism criteria in one of the eyes. Table 2 shows the descriptive characteristics of the participants who completed our study and of the eyes analyzed.

A similar temporal and inferior decentration of both the SV (0.41 ± 0.36 mm and 0.12 ± 0.15 mm, respectively) and the EDOF (0.45 ± 0.36 mm and 0.11 ± 0.17 mm, respectively) was observed with no statistically significant differences (*p* = 0.673, Wilcoxon test) between contact lenses.

### 3.2. Visual Acuity (VA) and High-Order Aberrations (HOAs)

The median and variance values of the monocular and binocular high- and low-contrast visual acuity with the single-vision (SV) and extended-depth-of-focus (EDOF) CLs are in Table 3. The paired comparison showed a statistically significant decrease of about 1 line in the monocular HCVA (*p* ≤ 0.005, Wilcoxon test) and about 1.5 lines in both the monocular and binocular LCVAs (*p* = 0.001, Wilcoxon test) with the EDOF. Although the difference between the SV and the EDOF in the binocular HCVA was statistically significant (*p* = 0.003, Wilcoxon test), the median difference corresponds to approximately two letters, which is not clinically relevant. Also, the monocular and binocular HCVAs of all subjects with the EDOF was 0.00 logMAR or better.

**Figure 4:**
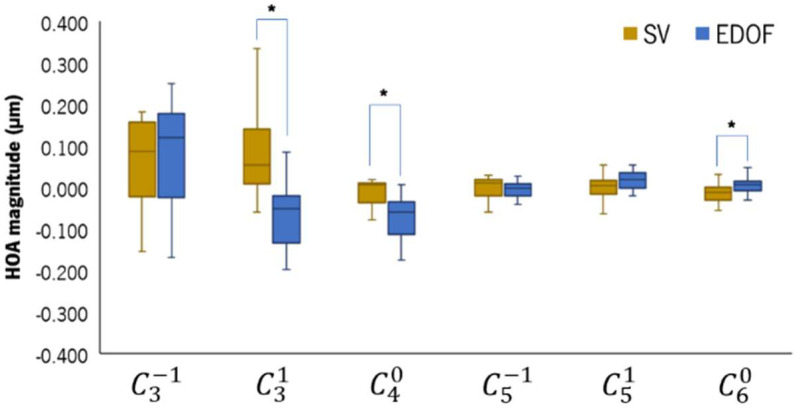
Boxplot of the high-order aberration coefficients obtained with the IRx3 Hartman–Shack aberrometer while patients were wearing the SV (light-brown) and the EDOF (blue) contact lenses. * Statistically significant differences with *p* ≤ 0.050, Wilcoxon test.

Figure 4 shows the HOAs measured with the SV and EDOF lenses on, represented in boxplots. The EDOF lens significantly changed the third-order horizontal coma ($C_{3}^{1}$) and the fourth-order ($C_{4}^{0}$) and sixth-order ($C_{6}^{0}$) spherical-like (SA) HOAs compared to the SV. The EDOF lens induced a median negative increase of 0.138 [0.012] µm in $C_{3}^{1}$ (*p* = 0.001) and of 0.076 [0.005] µm in $C_{4}^{0}$ (*p* = 0.002) compared to the SV. The median difference in $C_{6}^{0}$ between the EDOF and the SV was 0.018 [0.001] µm (*p* = 0.009). The total HOA RMS (not represented) did not show statistically significant differences between the two CLs.

### 3.3. Peripheral Refraction

The central (C) absolute refraction of the three refractive components (M, FT′ and FS′) was more negative with the EDOF compared to the SV: M (C)SV = −0.19 ± 0.30 D; M (C)EDOF = −0.39 ± 0.37 D; FT′ (C)SV = −0.11 ± 0.36 D; FT′ (C)EDOF = −0.19 ± 0.45 D; FS′ (C)SV = −0.26 ± 0.41 D; FS′ (C)EDOF = −0.59 ± 0.57 D. The differences between CLs were statistically significant for M (mean difference of −0.21 ± 0.37 D, 95% CI [−0.41; 0.00], *p* = 0.047) and FS′ (mean differences of −0.33 ± 0.41 D, 95% CI [−0.56; −0.10], *p* = 0.008).

**Figure 5:**
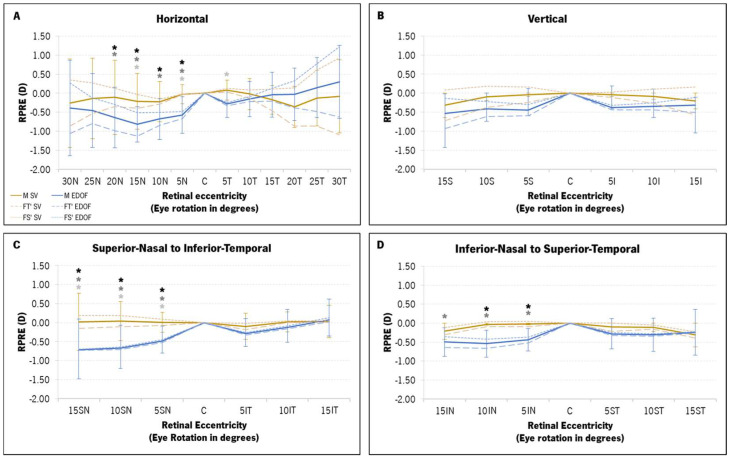
Mean relative refractive peripheral refractive error (RPRE) profile, in diopters (D), of the three components of refraction (M, FT′ and FS′) with the single-vision (SV, in light-brown) and the extended-depth-of-focus (EDOF, in blue) contact lenses for four retinal meridians: horizontal (**A**), vertical (**B**), superior–nasal (SN) to inferior–temporal (IT) (**C**) and inferior–nasal (IN) to superior–temporal (ST) (**D**). The M component is represented by a full line and darker colors, while FT′ and FS′ are represented in light colors (long-dashed lines and short-dashed lines, respectively). (*) statistically significant differences from the post hoc Bonferroni pairwise comparison between the two contact lenses at each point for the M component (black asterisk), FT′ (dark-gray asterisk) and FS′ (light-gray asterisk).

The SV and EDOF were compared in terms of relative peripheral refraction for the M, FT′ and FS′ components at each eccentric point. Figure 5 shows the profile of the RPRE of the horizontal (A), vertical (B), superior–nasal (SN) to inferior–temporal (IT) (C) and inferior–nasal (IN) to superior–temporal (ST) (D) retinal meridians (SV in light-brown and EDOF in blue). The three refractive components are represented as follows: full line (M component), long-dashed line (FT′) and short-dashed line (FS′).

A two-way ANOVA was executed to discern potential differences in the RPRE between the SV and the EDOF, according to eccentricity. Significant statistical variations surfaced between the two contact lenses (CLs) in M (*p* < 0.001), FT′ (*p* < 0.001) and FS′ (*p* < 0.001). The two-factor analysis, coupled with pairwise comparisons using post hoc Bonferroni corrections, showed statistically significant differences in all refractive components for nasal points 5N to 20N (Figure 5A), 5SN to 15SN (Figure 5C) and 5IN to 15IN (Figure 5D). The EDOF promoted a myopic shift in the RPRE across most retinal points for all refractive components, with a particularly pronounced shift in nasal eccentricities ranging from 5° to 20° (Figure 5A,C,D). Along the horizontal meridian, the EDOF appeared to induce a relative hyperopic shift beyond the temporal 10° mark (15T to 30T, Figure 5A), albeit without statistically significant differences compared to the SV. The M and FT′ RPRE of the SV exhibited a more substantial myopic shift with the EDOF compared to the FS′. The maximum myopic shift induced by the EDOF lenses in the M, FT′ and FS′ components was observed at the 15SN point (Figure 5C): mean difference of −0.73 ± 0.77 D in M (95% CI [−1.15; −0.32], *p* = 0.001); mean difference of −0.89 ± 0.79 D in FT′ (95% CI [−1.05; −0.02], *p* < 0.001); and −0.58 ± 0.87 D in FS′ (95% CI [−1.02; −0.15], *p* < 0.001).

### 3.4. Global-Flash mfERG Response

The mean pupil diameter before the gf-mfERG recordings was 5.35 ± 0.53 mm with the SV lens and 5.36 ± 0.51 mm with the EDOF lens, and after the gf-mfERG recordings, it was 5.35 ± 0.50 mm with the SV lens and 5.38 ± 0.52 mm with the EDOF lens. There were no statistically significant differences within recordings (before versus after gf-mfERG recording, *p* ≥ 0.822) or between the SV and the EDOF (*p* ≥ 0.745).

The median and interquartile distance [IQR] of the DC and IC response densities from gf-mfERG of each CL for each retinal area evaluated (total retinal area, eccentric areas and quadrants) are shown in Table 4 and Table 5, respectively.

No statistically significant differences were observed in the implicit time of the DC and IC peaks between the SV and the EDOF in the total retinal response, neither between eccentric areas nor quadrants.

A two-way ANOVA showed that the response density of both the DC and the IC decreased significantly with eccentricity (Table 4 and Table 5). Additionally, the response density of the IC component was significantly different between retinal quadrants (Table 5). The post hoc Bonferroni corrections showed that the significant response density decrease of the two gf-mfERG components with eccentricity was not statistically significant only between the para-macula and the periphery with the EDOF lens (*p* = 0.065). Regarding the analysis by quadrants, a two-way ANOVA showed statistically significant differences in the IC response density between the two CLs (Table 5). The post hoc Bonferroni pairwise comparisons showed statistically significant differences between the INQ and the SNQ and between the SNQ and the ITQ with the SV (*p* = 0.048 and *p* = 0.006, respectively) and the EDOF (*p* = 0.016 and *p* = 0.017, respectively).

**Figure 6:**
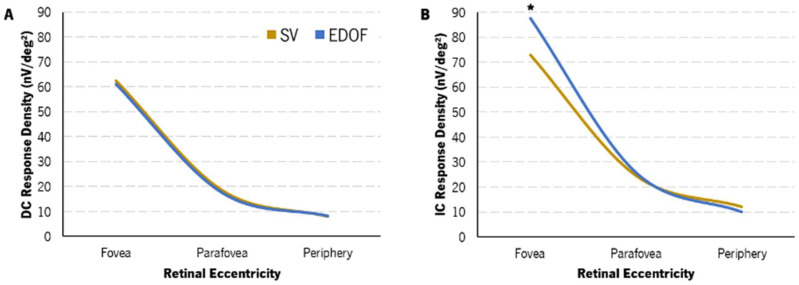
Change in DC (**A**) and IC (**B**) response density with eccentricity with SV (light-brown) and EDOF (blue) contact lenses. Variance and IQR are not represented for clarity. EDOF showed a statistically significantly higher IC response density in the fovea compared to SV (*).

Regarding the comparison of response density between the two CLs, no differences were found in the DC component in any retinal area. A post hoc Bonferroni pairwise analysis showed a statistically significant median difference of 13.31 [23.88–3.85] nV/deg^2^ in the response density of the IC component (*p* = 0.036, Table 5). These can also be seen in Figure 6B of the IC component depicted with retinal eccentricity.

**Figure 7:**
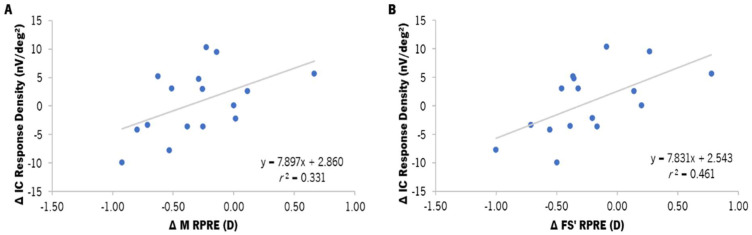
Correlation between the differences in the response density of the induced component (IC, in nV/deg^2^) in the para-macula and the median value of the differences in the M component of the RPRE (**A**) and the sagittal foci (FS′) of the RPRE (**B**) in the para-macular retinal area (median from the 5° to the 20° nasal and temporal points of the horizontal meridian), according to the areas established for gf-mfERG analysis. ∆ refers to the difference between EDOF and SV (EDOF–SV) values. *r*^2^ refers to the determination coefficient of Spearman’s correlation. The gray line represents the best-fit line adjusted to the data.

The analysis of the correlation between the changes in the RPRE and the changes in the response densities of the gf-mfERG response was conducted by Spearman’s correlation. The variation in the response density of the IC component in the para-macula showed statistically significant correlations with the M (*r* = 0.575, *p* = 0.025) and FS′ (*r* = 0.679, *p* = 0.005) components of the para-macula (Figure 7A and Figure 7B, respectively). Nevertheless, as shown in Figure 7, this correlation was limited by the amount of data and had a moderate dispersion.

## 4. Discussion

This is the first study evaluating the optical and retinal performance of a commercially available extended-depth-of-focus (EDOF) contact lens (CL) used for myopia management, compared with a material-matched single-vision CL.

The EDOF lens induced a significant negative increase in the fourth-order spherical HOA and decreased both the HCVA (one line) and the LCVA (two lines). Beyond the relative myopic shift of the peripheral refractive profile compared to the single-vision CL (SV), after 30 min of lens wear, the EDOF lenses showed a significant enhancement in the response of the innermost retinal layers at the fovea, while the response of the remaining retinal regions was similar to the SV CL. The response density of the induced component (IC) of the para-macula with the EDOF decreased as the myopic shift was higher.

Most myopic eyes tend to have relative peripheral hyperopia [41]. Several studies have reported that myopia control strategies decrease the rate of eye growth at the expense of inducing an astigmatic myopic shift in the peripheral retina, concerning the typical relative peripheral defocus of myopic eyes [32]. Compared to the on-axis point, this will produce a greater myopic shift in the focalization across the peripheral retina than that produced with a spherical or aspherical single-vision lens. The EDOF lens induced a myopic shift over most of the retinal points that was greater in the tangential foci than in the sagittal foci [32]. However, the RPRE beyond the 10° mark of the temporal retina with the EDOF lens showed a hyperopic shift relative to the SV lens. In an EDOF lens, the light is distributed across an extended range that dictates the range of distances over which the lens might provide a clear image, with no discrete zones of constant power. The light that strikes the retina within this range is in focus and contributes to the perception of an image. In this study, an EDOF lens of negative spherical aberration was used. In this case, the rays passing through the edge of the lens were hyperopic relative to the rays passing through the center of the lens. Together with the slight temporal decentration of the optical center of the lens of 0.45 ± 0.36 mm, this exacerbated that effect. The impact of the EDOF lens decentration needs a more comprehensive analysis in future studies.

The EDOF lens increased the retinal response density in the central area (fovea) and decreased or did not change it in the remaining regions (perifoveal concentric areas and quadrants) compared to the SV lens. This suggests that, in general, the inner retina is sensitive to more positive (myopic) defocus, as previously found in other studies [26,27]. Also, the retinal response decreases with higher levels of defocus in both the inner and outer layers [26]. Studies have reported a higher decrease in the retinal response in the parafoveal and peripheral regions [26,27]. The present study has shown similar results: The EDOF lens promoted the myopic shift (positive defocus) of the peripheral refraction and reduced the gf-mfERG response in the para-macular and peripheral areas. This effect was greater in the periphery, where the response density of the IC component was lower, although not significant.

On the other hand, the foveal response with the EDOF lens was significantly more vigorous in the inner retina (higher foveal response density in the IC component), while the outer retinal response was unchanged. This is probably due to the myopic shift observed in the overall refractive profile, potentially induced by the progressive decrease in negative power in the central 3 mm diameter zone of the EDOF lens. Ho et al. observed similar behavior in the central region with 2.00 D myopic defocus, although there was a decrease in the central retinal response with higher levels of defocus [26]. A study by Fung et al. using dual-focus contact lenses with concentric in-focus and positive defocus rings showed an enhancement in the retinal response in the central retinal region and in the para-macular regions. Although this enhancement was observed in both the DC and IC components, only that originating from the inner layers (the IC component) was significant with a dual-focus contact lens with +7.50 D addition [28]. Although the results of the present study may partially agree with those of Fung et al., they cannot be fully comparable due to the differences in lens designs and measurement conditions.

Several studies on myopia development and progression in children have shown that the central retinal function shows a subclinical decrease before myopia development, considering the central retinal function a potential myogenic factor and reference for myopia development [23,42,43]. When investigating the characteristics of retinal activity in children with early myopia development, Li et al. observed a subclinical reduction in the central IC amplitude (inner retina) 1 year before myopia onset, compared to children who remained emmetropes. The central IC response density under the 49% contrast of the initial visit was the only electrophysiological parameter to significantly correlate to the refractive error change over a year [23]. These findings, together with the foveal IC response density increase observed with the EDOF lens in the present study, suggest that this myopia management corrective tool may improve the retinal activity of myopic eyes, whose central electrical response is lower compared to emmetropes. In a similar way, blind-spot stimulation with a blue light stimulus increased the amplitude of the retinal response in myopic eyes, while non-myopic eyes did not experience any effect [16].

Following the fovea, the para-macula (comprising the parafovea and perifovea) is the retinal region that influences the foveal image the most [44]. A myopic defocus in this area could improve the perception of the foveal image [45,46] and, consequently, potentiate the response of the innermost retinal cells that contribute to the IC component of the gf-mfERG response. Additionally, the EDOF lens induced an absolute decrease in spherical aberration compared with the SV. Still, the foveal response density of the IC component was higher with the EDOF lens. A study by Panorgias et al. [47] showed that spherical aberration changes the retinal response more than a defocus of the same magnitude. Together, these considerations suggest that the induction of more negative spherical aberration, combined with the changes in para-macular response to defocus, still allow for the enhancement of the inner foveal retinal activity response, since the retinal response might be due to a summative effect of defocus and spherical aberration [48].

Another perspective should be considered when discussing the increase in response density of the foveal innermost layers with EDOF lenses. The response of these layers is influenced by the interactions between bipolar, amacrine and ganglion cells in the inner plexiform layer. Currently, a very peculiar type of ganglion cell has been the subject of study because it is intrinsically photosensitive and has the ability to communicate with the retina in a retrograde manner [49]. Some researchers hypothesize that their electrophysiological characteristics allow these cells to play a role not only in the regulation of circadian rhythms and the perception and discrimination of brightness and contrast but also in the spatial interpretation of visual information, such as defocus detection [50]. As such, the results of this study may suggest a possible feedback mechanism in the inner retina, involving inner cells that respond differently to more positive defocus towards the central fovea produced by the EDOF lens.

The present study has some limitations. Although the HCVA obtained with both lenses was satisfactory, the inclusion criteria account for low astigmatism (up to 1.00 D). For this, some participants showed uncorrected cylindrical refraction since the CLs did not have toric geometry. However, low levels of astigmatism did not induce significant changes in the electrophysiological response of the retina in previous studies [47,51]. One might have thought that the differences in HCVA could also be overcome by over-refraction in order to guarantee a more similar visual acuity between the EDOF and the SV. However, according to the fitting guide for these myopia management contact lenses, over-refraction should be performed if the visual acuity is inferior to 20/25 ft in the initial fit, which did not occur in any subject. Also, considering the use of an EDOF lens based on over-refraction could result in the use of EDOF and SV lenses with distinct effective powers, consequently decreasing the comparison between results. Furthermore, it is more likely that the decrease in HCVA and LCVA is due to the high-order aberrations induced by the EDOF lens’s design.

All the ERG measurements should be performed under pupil dilations to reduce the influence of pupil fluctuations and accommodation during recordings [35]. However, in the present study, the gf-mfERG was obtained with non-dilated pupils to evaluate the response under physiological conditions. The use of these types of CLs showed a change in accommodative capacity that may be imperative for the successful retention rate of eye elongation [14,52]. Pupil dilation would allow for stronger electrophysiological signals, but even under non-dilated conditions, the activity was robust enough to measure the effects observed.

For future studies, the assessment of the visual contrast sensitivity function may be considered to study the impact of contrast perception on electrophysiological changes with myopia management contact lenses. Also, it would be imperative to analyze the behavior of other myopia management CLs available on the market, as well as other myopia retention methods (ophthalmic lenses and other innovative methods). Since the target population for myopia management is children, it is crucial to study how their retinas respond to different optical treatments while tracking the changes in axial length over time.

Nowadays, there are several myopia management approaches—environmental (outdoor–indoor activities), pharmacological, and optical solutions—resulting in different retention rates, rebound effects and risks. When choosing an appropriate and personalized strategy (either a single strategy or combined strategies), inter-subject variability characteristics, such as age, ethnicity, retinal shape, rate of myopia progression, individual outdoor and indoor routines and risks, should be taken into account. The specific EDOF lens design used in the present study might potentially mitigate axial elongation through enhancement in the foveal inner layer’s activity, while the para-macula may also play a crucial role in the mechanisms to control eye elongation, as supported and suggested in previous studies. Therefore, this study reinforces the need for understanding the effect of different myopia management strategies on the retinal response and the mechanisms involved in controlling eye growth. This suggests that retinal electrical activity can also be a valuable aid and a potential biomarker for selecting the most suitable myopia management strategies based on individual factors.

## 5. Conclusions

The electrophysiological results with a myopia management CL reinforce previously reported findings that the retina recognizes and responds differently to defocus. The EDOF lens induced an improvement in the foveal inner layers’ response in young adults, potentially due to a summative effect of the negative spherical aberration through-focus design and the change in peripheral refraction. This study supports the hypothesis that the para-macula may have an important role in successful mechanisms to control myopia progression and that the retina is sensitive to the EDOF design. Since this CL design showed considerable retention rates in axial elongation in children, the results of the present study suggest that this might be possible by potentiating the retinal response of some retinal areas while maintaining optimally corrected on-axis refraction.

## Figures and Tables

**Table 1:** Contact lenses’ information.

	SV	EDOF
Optical design	Spherical	Extended-depth-of-focus
Substitution	Monthly	
Material	Filcon 5B (Silicone hydrogel)	
Hydration (%)	75	
DK/t	50	
Diameter (mm)	13.00–16.00 (0.50 steps)	13.50–15.50 (0.50 steps)
Radius (mm)	6.80–9.80 (0.30 steps)	7.10–9.80 (0.30 steps)

SV—single-vision contact lens, control condition; EDOF—extended-depth-of-focus contact lens, test condition; DK/t—oxygen transmissibility; mm—millimeters.

**Table 2:** Sample characteristics of the participants considered for data analysis (average ± SD).

Eyes (N)	16
Gender (no. of eyes)	3 male/13 female
Age (years)	27.5 ± 5.7 [18.9–38.4]
M (D)	−3.09 ± 1.50
J0 (D)	0.03 ± 0.16
J45 (D)	−0.03 ± 0.15
k_flat_ (mm)	7.86 ± 0.21
k_steep_ (mm)	7.70 ± 0.22
k_mean_ (mm)	7.78 ± 0.21
HDVI (mm)	11.70 ± 0.25

M—spherical equivalent; D—diopters; J0—cartesian astigmatism; J45—oblique astigmatism; k—corneal radius of curvature; mm—millimeters; HDVI—horizontal diameter of the visible iris.

**Table 3:** Monocular and binocular high-contrast (HCVA) and low-contrast (LCVA) visual acuity (median [variance] in logMAR units) with SV and EDOF CLs.

	HCVA (LogMAR)		LCVA (LogMAR)	
	Monocular	Binocular	Monocular	Binocular
SV	−0.10 [0.01]	−0.13 [0.00]	0.16 [0.02]	0.06 [0.00]
EDOF	−0.03 [0.01]	−0.08 [0.01]	0.34 [0.01]	0.21 [0.01]
*p*-value	0.005 *	0.003 *	0.001 *	0.001 *

HCVA—high-contrast visual acuity; LCVA—low-contrast visual acuity; SV—single-vision contact lens; EDOF—extended-depth-of-focus contact lens; LogMAR—Logarithm of the Minimum Angle of Resolution; *p*-values from the paired comparisons between lens designs; (*) statistically significant differences with the Wilcoxon test.

**Table 4:** Direct component (DC) response density in nV/deg^2^ (median [IQR]) of each CL (SV and EDOF) at different retinal areas.

DC (nV/deg^2^)		SV	EDOF	*p*
SUM		9.63 [11.14–7.83]	9.71 [11.18–8.81]	0.776
Eccentricity	Fovea	62.50 [73.87–53.15]	61.08 [74.42–50.37]	0.703
	Para-macula	18.62 [22.26–15.26]	17.62 [19.78–15.72]	0.819
	Periphery	8.11 [9.28–7.28]	8.31 [9.85–7.32]	0.964
	*p*	<0.001 *		
Quadrants	INQ	9.64 [13.89–8.48]	10.55 [11.95–9.32]	0.986
	SNQ	8.28 [10.41–7.34]	9.05 [10.53–8.15]	0.667
	STQ	9.22 [12.37–7.83]	9.79 [10.64–7.95]	0.663
	ITQ	10.75 [12.24–9.05]	11.03 [12.69–8.65]	0.858
	*p*	0.363		

DC—direct component; nV/deg^2^—nanoVolts per square degree; SV—single-vision contact lens; EDOF—extended-depth-of-focus contact lens (in blue); INQ—inferior–nasal quadrant; SNQ—superior–nasal quadrant; STQ—superior–temporal quadrant; ITQ—inferior–temporal quadrant; *p*-values indicate the significance of the two-way ANOVA for eccentricity and quadrants and the differences by post hoc Bonferroni corrections between the two CLs; * *p*-values < 0.050, showing statistically significant differences.

**Table 5:** Induced component (IC) response density in nV/deg^2^ (median [IQR]) of each CL (SV and EDOF) at different retinal areas.

IC (nV/deg^2^)		SV	EDOF	*p*
SUM		11.13 [11.86–8.25]	11.07 [11.95–7.29]	0.069
Eccentricity	Fovea	73.00 [99.58–64.77]	87.62 [100.35–70.00]	0.036 *
	Para-macula	24.52 [30.26–21.40]	25.79 [30.48–21.91]	0.943
	Periphery	12.03 [12.86–10.30]	10.04 [12.63–9.21]	0.930
	*p*	<0.001 *		
Quadrants	INQ	11.53 [14.58–8.82]	11.80 [14.15–8.59]	0.811
	SNQ	9.79 [10.86–6.95]	7.37 [10.20–6.41]	0.492
	STQ	11.22 [13.74–8.93]	10.86 [13.59–8.10]	0.673
	ITQ	12.31 [15.59–8.49]	10.51 [13.74–8.95]	0.285
	*p*	0.018 *		

IC—induced component; nV/deg^2^—nanoVolts per square degree; SV—single-vision contact lens; EDOF—extended-depth-of-focus contact lens (in blue); INQ—inferior–nasal quadrant; SNQ—superior–nasal quadrant; STQ—superior–temporal quadrant; ITQ—inferior–temporal quadrant; *p*-values indicate the significance of the two-way ANOVA for eccentricity and quadrants and the differences by post hoc Bonferroni corrections between the two CLs; * *p*-values < 0.050, showing statistically significant differences.

## Data Availability

The raw data supporting the conclusions of this article will be made available by the authors on request.
